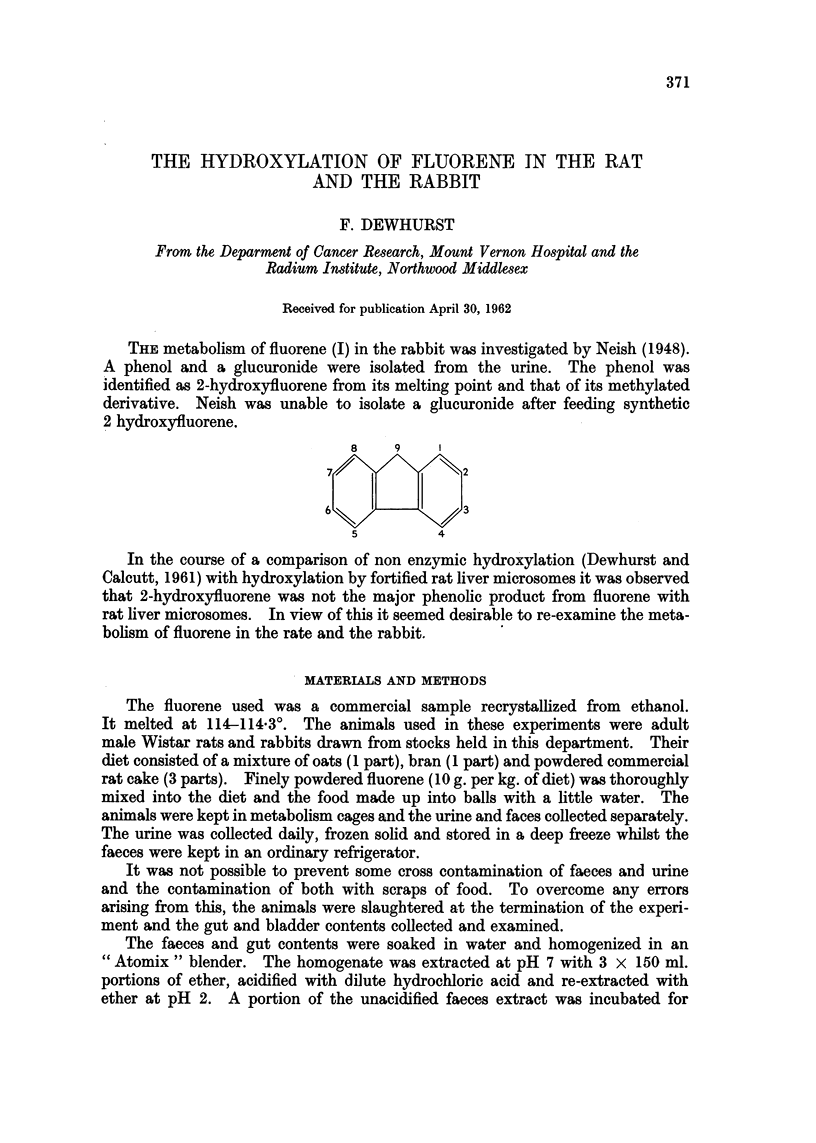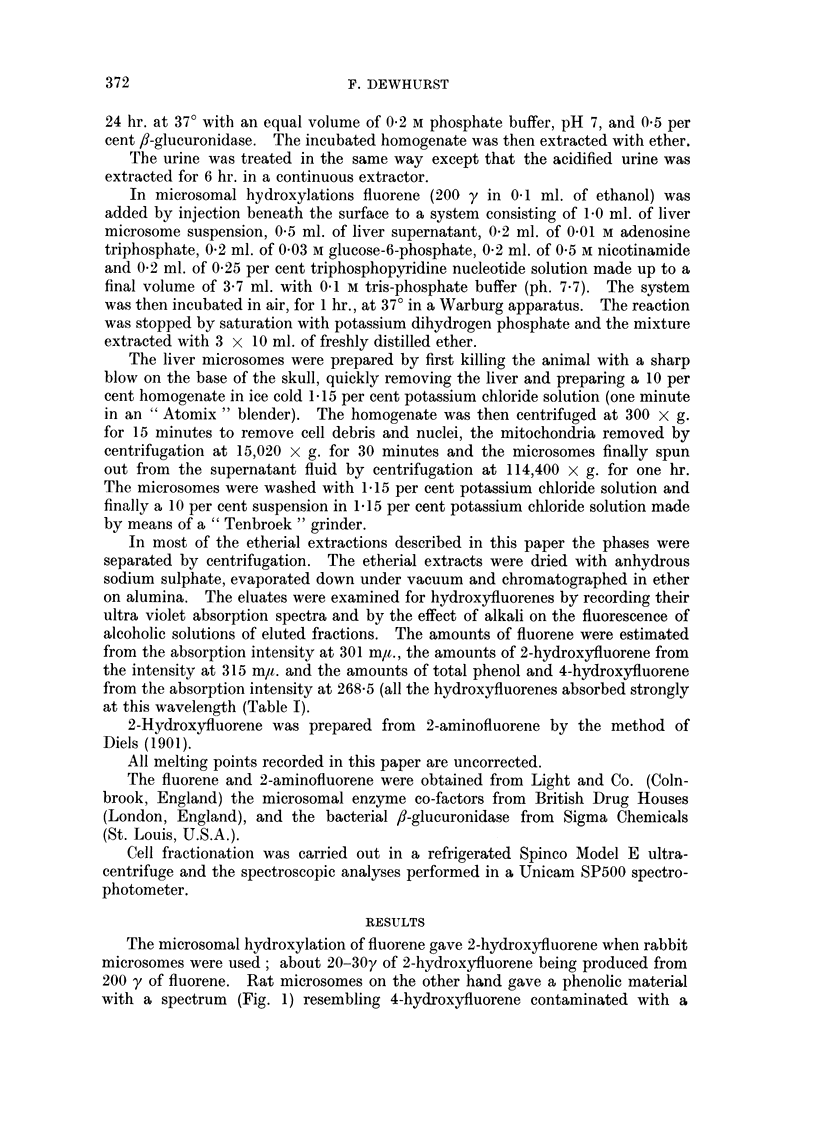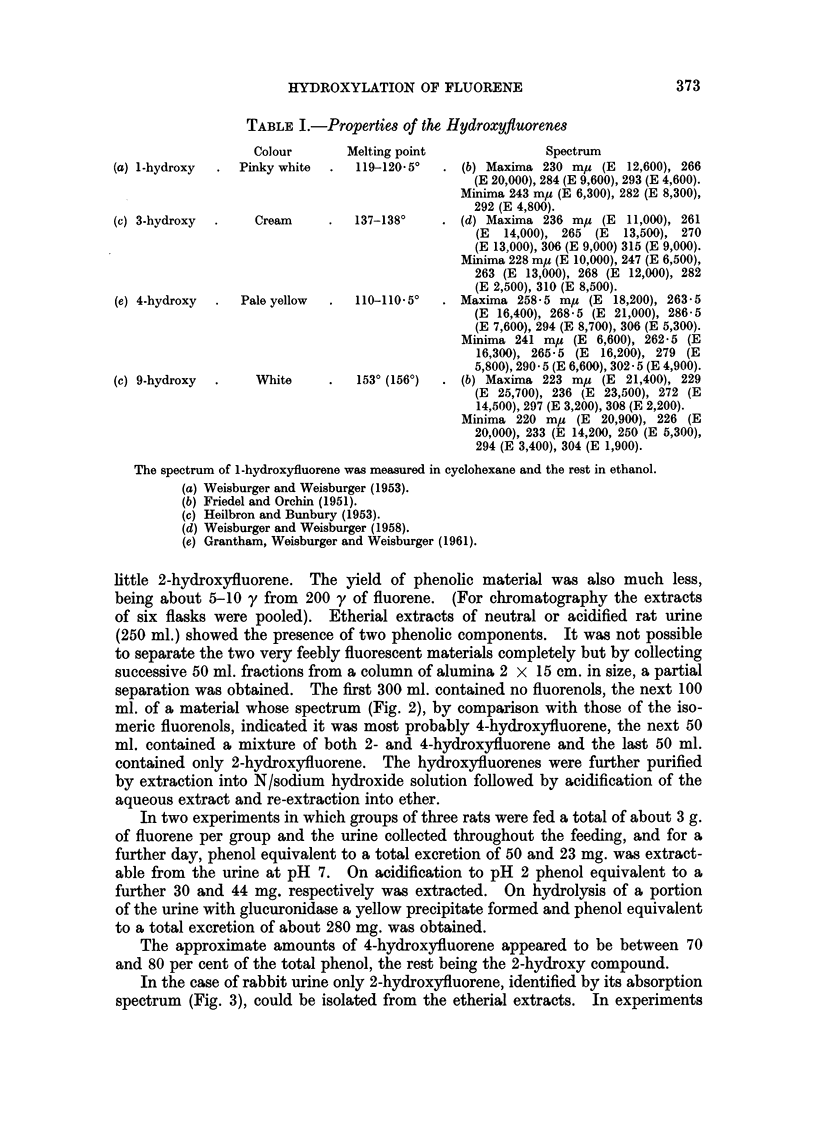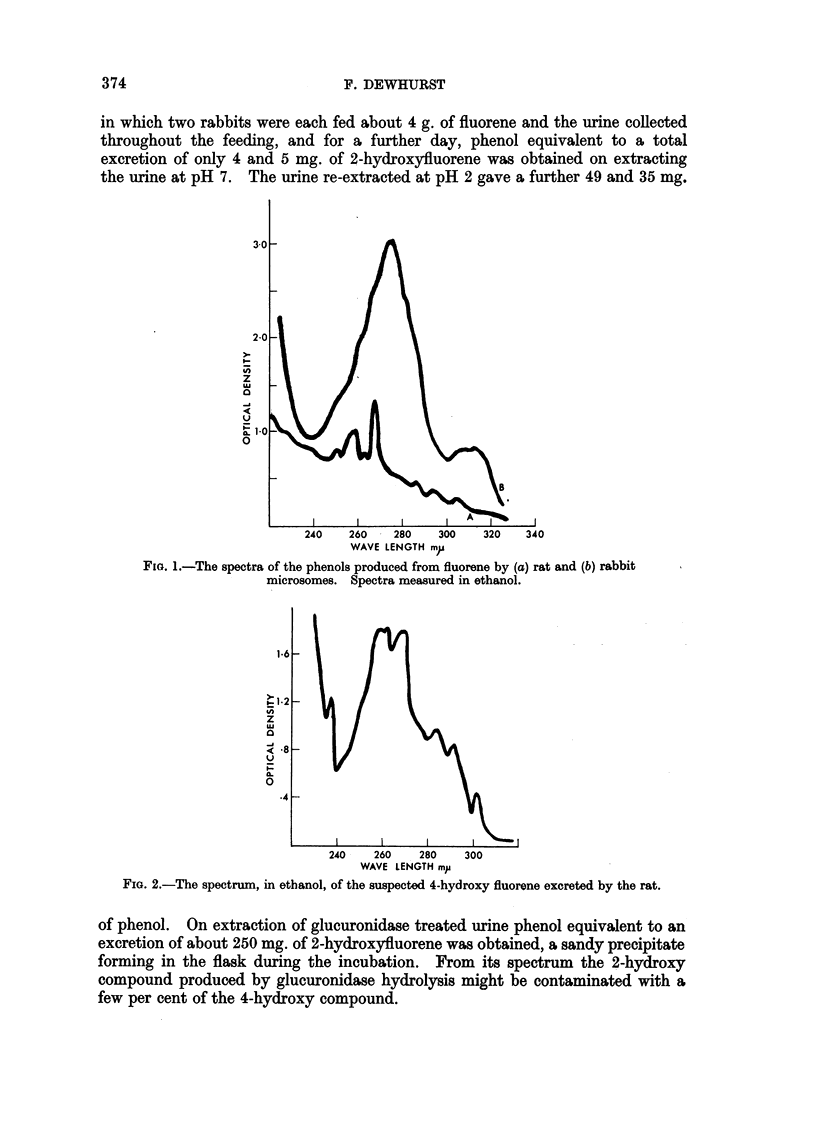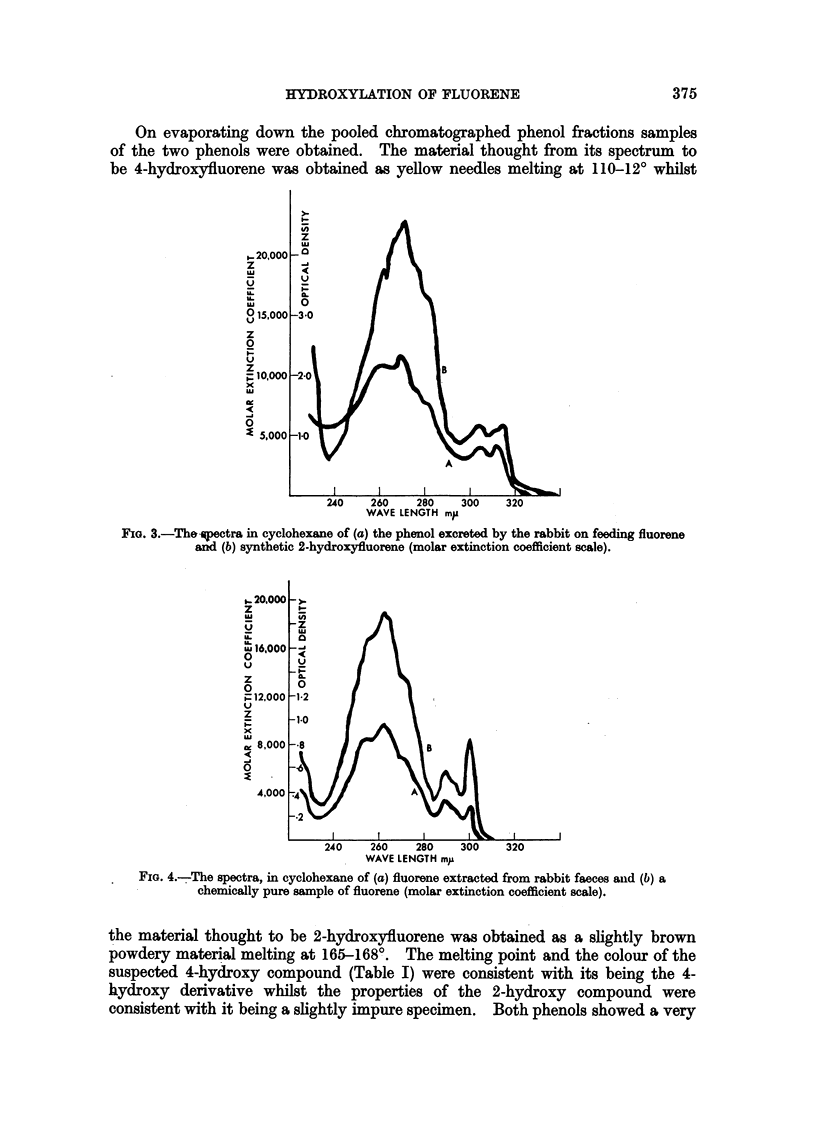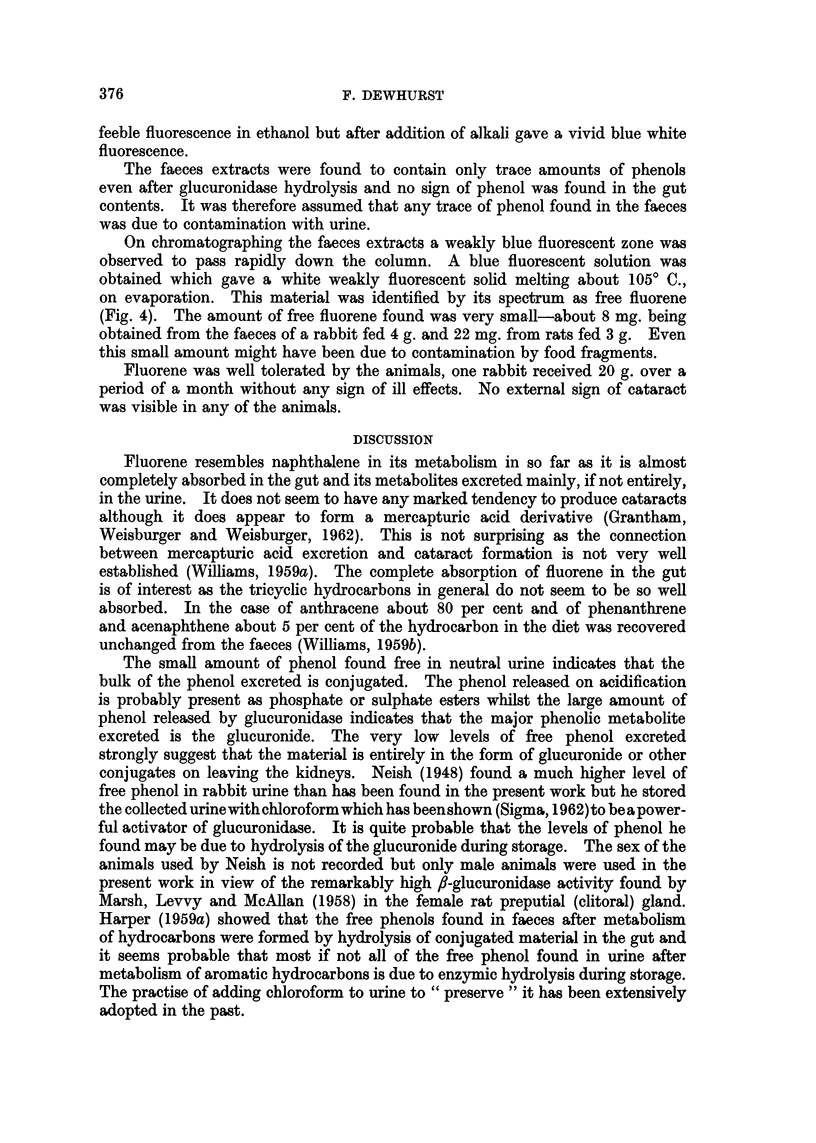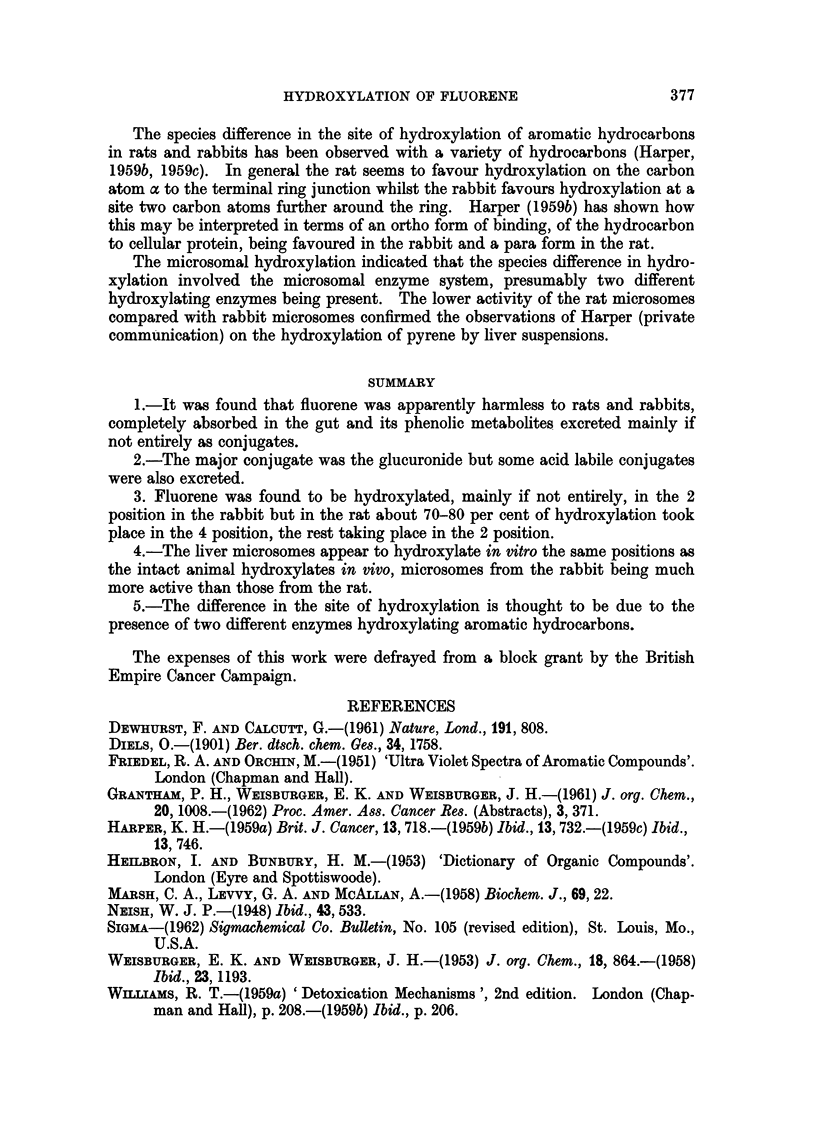# The Hydroxylation of Fluorene in the Rat and the Rabbit

**DOI:** 10.1038/bjc.1962.42

**Published:** 1962-06

**Authors:** F. Dewhurst


### THE HYDROXYLATION OF FLUORENE IN THE RAT AND THE RABBIT

F. DEWHURST

*From the Deparment of Cancer Research, Mount Vernon Hospital and the Radium Institute, Northwood Middlesex*

Received for publication April 30, 1962

THE metabolism of fluorene (I) in the rabbit was investigated by Neish (1948). A phenol and a glucuronide were isolated from the urine. The phenol was identified as 2-hydroxyfluorene from its melting point and that of its methylated derivative. Neish was unable to isolate a glucuronide after feeding synthetic 2 hydroxyfluorene.

In the course of a comparison of non enzymic hydroxylation (Dewhurst and Calcutt, 1961) with hydroxylation by fortified rat liver microsomes it was observed that 2-hydroxyfluorene was not the major phenolic product from fluorene with rat liver microsomes. In view of this it seemed desirable to re-examine the metabolism of fluorene in the rate and the rabbit.

### MATERIALS AND METHODS

The fluorene used was a commercial sample recrystallized from ethanol. It melted at 114–114·3°. The animals used in these experiments were adult male Wistar rats and rabbits drawn from stocks held in this department. Their diet consisted of a mixture of oats (1 part), bran (1 part) and powdered commercial rat cake (3 parts). Finely powdered fluorene (10 g. per kg. of diet) was thoroughly mixed into the diet and the food made up into balls with a little water. The animals were kept in metabolism cages and the urine and faces collected separately. The urine was collected daily, frozen solid and stored in a deep freeze whilst the faeces were kept in an ordinary refrigerator.

It was not possible to prevent some cross contamination of faeces and urine and the contamination of both with scraps of food. To overcome any errors arising from this, the animals were slaughtered at the termination of the experiment and the gut and bladder contents collected and examined.

The faeces and gut contents were soaked in water and homogenized in an "Atomix" blender. The homogenate was extracted at pH 7 with 3 × 150 ml. portions of ether, acidified with dilute hydrochloric acid and re-extracted with ether at pH 2. A portion of the unacidified faeces extract was incubated for

24 hr. at 37° with an equal volume of 0·2 M phosphate buffer, pH 7, and 0·5 per cent $\beta$-glucuronidase. The incubated homogenate was then extracted with ether.

The urine was treated in the same way except that the acidified urine was extracted for 6 hr. in a continuous extractor.

In microsomal hydroxylations fluorene (200 $\gamma$ in 0·1 ml. of ethanol) was added by injection beneath the surface to a system consisting of 1·0 ml. of liver microsome suspension, 0·5 ml. of liver supernatant, 0·2 ml. of 0·01 M adenosine triphosphate, 0·2 ml. of 0·03 M glucose-6-phosphate, 0·2 ml. of 0·5 M nicotinamide and 0·2 ml. of 0·25 per cent triphosphopyridine nucleotide solution made up to a final volume of 3·7 ml. with 0·1 M tris-phosphate buffer (ph. 7·7). The system was then incubated in air, for 1 hr., at 37° in a Warburg apparatus. The reaction was stopped by saturation with potassium dihydrogen phosphate and the mixture extracted with 3 × 10 ml. of freshly distilled ether.

The liver microsomes were prepared by first killing the animal with a sharp blow on the base of the skull, quickly removing the liver and preparing a 10 per cent homogenate in ice cold 1·15 per cent potassium chloride solution (one minute in an " Atomix " blender). The homogenate was then centrifuged at 300 × g. for 15 minutes to remove cell debris and nuclei, the mitochondria removed by centrifugation at 15,020 × g. for 30 minutes and the microsomes finally spun out from the supernatant fluid by centrifugation at 114,400 × g. for one hr. The microsomes were washed with 1·15 per cent potassium chloride solution and finally a 10 per cent suspension in 1·15 per cent potassium chloride solution made by means of a " Tenbroek " grinder.

In most of the etherial extractions described in this paper the phases were separated by centrifugation. The etherial extracts were dried with anhydrous sodium sulphate, evaporated down under vacuum and chromatographed in ether on alumina. The eluates were examined for hydroxyfluorenes by recording their ultra violet absorption spectra and by the effect of alkali on the fluorescence of alcoholic solutions of eluted fractions. The amounts of fluorene were estimated from the absorption intensity at 301 m$\mu$., the amounts of 2-hydroxyfluorene from the intensity at 315 m$\mu$. and the amounts of total phenol and 4-hydroxyfluorene from the absorption intensity at 268·5 (all the hydroxyfluorenes absorbed strongly at this wavelength (Table I).

2-Hydroxyfluorene was prepared from 2-aminofluorene by the method of Diels (1901).

All melting points recorded in this paper are uncorrected.

The fluorene and 2-aminofluorene were obtained from Light and Co. (Colnbrook, England) the microsomal enzyme co-factors from British Drug Houses (London, England), and the bacterial $\beta$-glucuronidase from Sigma Chemicals (St. Louis, U.S.A.).

Cell fractionation was carried out in a refrigerated Spinco Model E ultracentrifuge and the spectroscopic analyses performed in a Unicam SP500 spectrophotometer.

### RESULTS

The microsomal hydroxylation of fluorene gave 2-hydroxyfluorene when rabbit microsomes were used ; about 20–30$\gamma$ of 2-hydroxyfluorene being produced from 200 $\gamma$ of fluorene. Rat microsomes on the other hand gave a phenolic material with a spectrum (Fig. 1) resembling 4-hydroxyfluorene contaminated with a

TABLE I.—*Properties of the Hydroxyfluorenes*

| | Colour | Melting point | Spectrum |
|---|---|---|---|
| (*a*) 1-hydroxy | Pinky white | 119–120·5° | (*b*) Maxima 230 mμ (E 12,600), 266 (E 20,000), 284 (E 9,600), 293 (E 4,600). Minima 243 mμ (E 6,300), 282 (E 8,300), 292 (E 4,800). |
| (*c*) 3-hydroxy | Cream | 137–138° | (*d*) Maxima 236 mμ (E 11,000), 261 (E 14,000), 265 (E 13,500), 270 (E 13,000), 306 (E 9,000) 315 (E 9,000). Minima 228 mμ (E 10,000), 247 (E 6,500), 263 (E 13,000), 268 (E 12,000), 282 (E 2,500), 310 (E 8,500). |
| (*e*) 4-hydroxy | Pale yellow | 110–110·5° | Maxima 258·5 mμ (E 18,200), 263·5 (E 16,400), 268·5 (E 21,000), 286·5 (E 7,600), 294 (E 8,700), 306 (E 5,300). Minima 241 mμ (E 6,600), 262·5 (E 16,300), 265·5 (E 16,200), 279 (E 5,800), 290·5 (E 6,600), 302·5 (E 4,900). |
| (*c*) 9-hydroxy | White | 153° (156°) | (*b*) Maxima 223 mμ (E 21,400), 229 (E 25,700), 236 (E 23,500), 272 (E 14,500), 297 (E 3,200), 308 (E 2,200). Minima 220 mμ (E 20,900), 226 (E 20,000), 233 (E 14,200, 250 (E 5,300), 294 (E 3,400), 304 (E 1,900). |

The spectrum of 1-hydroxyfluorene was measured in cyclohexane and the rest in ethanol.

(*a*) Weisburger and Weisburger (1953).
(*b*) Friedel and Orchin (1951).
(*c*) Heilbron and Bunbury (1953).
(*d*) Weisburger and Weisburger (1958).
(*e*) Grantham, Weisburger and Weisburger (1961).

little 2-hydroxyfluorene. The yield of phenolic material was also much less, being about 5–10 γ from 200 γ of fluorene. (For chromatography the extracts of six flasks were pooled). Etherial extracts of neutral or acidified rat urine (250 ml.) showed the presence of two phenolic components. It was not possible to separate the two very feebly fluorescent materials completely but by collecting successive 50 ml. fractions from a column of alumina 2 × 15 cm. in size, a partial separation was obtained. The first 300 ml. contained no fluorenols, the next 100 ml. of a material whose spectrum (Fig. 2), by comparison with those of the isomeric fluorenols, indicated it was most probably 4-hydroxyfluorene, the next 50 ml. contained a mixture of both 2- and 4-hydroxyfluorene and the last 50 ml. contained only 2-hydroxyfluorene. The hydroxyfluorenes were further purified by extraction into N/sodium hydroxide solution followed by acidification of the aqueous extract and re-extraction into ether.

In two experiments in which groups of three rats were fed a total of about 3 g. of fluorene per group and the urine collected throughout the feeding, and for a further day, phenol equivalent to a total excretion of 50 and 23 mg. was extractable from the urine at pH 7. On acidification to pH 2 phenol equivalent to a further 30 and 44 mg. respectively was extracted. On hydrolysis of a portion of the urine with glucuronidase a yellow precipitate formed and phenol equivalent to a total excretion of about 280 mg. was obtained.

The approximate amounts of 4-hydroxyfluorene appeared to be between 70 and 80 per cent of the total phenol, the rest being the 2-hydroxy compound.

In the case of rabbit urine only 2-hydroxyfluorene, identified by its absorption spectrum (Fig. 3), could be isolated from the etherial extracts. In experiments in which two rabbits were each fed about 4 g. of fluorene and the urine collected throughout the feeding, and for a further day, phenol equivalent to a total excretion of only 4 and 5 mg. of 2-hydroxyfluorene was obtained on extracting the urine at pH 7. The urine re-extracted at pH 2 gave a further 49 and 35 mg.

Fig. 1.—The spectra of the phenols produced from fluorene by (*a*) rat and (*b*) rabbit microsomes. Spectra measured in ethanol.

Fig. 2.—The spectrum, in ethanol, of the suspected 4-hydroxy fluorene excreted by the rat.

of phenol. On extraction of glucuronidase treated urine phenol equivalent to an excretion of about 250 mg. of 2-hydroxyfluorene was obtained, a sandy precipitate forming in the flask during the incubation. From its spectrum the 2-hydroxy compound produced by glucuronidase hydrolysis might be contaminated with a few per cent of the 4-hydroxy compound.

On evaporating down the pooled chromatographed phenol fractions samples of the two phenols were obtained. The material thought from its spectrum to be 4-hydroxyfluorene was obtained as yellow needles melting at 110–12° whilst

FIG. 3.—The spectra in cyclohexane of (*a*) the phenol excreted by the rabbit on feeding fluorene and (*b*) synthetic 2-hydroxyfluorene (molar extinction coefficient scale).

FIG. 4.—The spectra, in cyclohexane of (*a*) fluorene extracted from rabbit faeces and (*b*) a chemically pure sample of fluorene (molar extinction coefficient scale).

the material thought to be 2-hydroxyfluorene was obtained as a slightly brown powdery material melting at 165–168°. The melting point and the colour of the suspected 4-hydroxy compound (Table I) were consistent with its being the 4-hydroxy derivative whilst the properties of the 2-hydroxy compound were consistent with it being a slightly impure specimen. Both phenols showed a very feeble fluorescence in ethanol but after addition of alkali gave a vivid blue white fluorescence.

The faeces extracts were found to contain only trace amounts of phenols even after glucuronidase hydrolysis and no sign of phenol was found in the gut contents. It was therefore assumed that any trace of phenol found in the faeces was due to contamination with urine.

On chromatographing the faeces extracts a weakly blue fluorescent zone was observed to pass rapidly down the column. A blue fluorescent solution was obtained which gave a white weakly fluorescent solid melting about 105° C., on evaporation. This material was identified by its spectrum as free fluorene (Fig. 4). The amount of free fluorene found was very small—about 8 mg. being obtained from the faeces of a rabbit fed 4 g. and 22 mg. from rats fed 3 g. Even this small amount might have been due to contamination by food fragments.

Fluorene was well tolerated by the animals, one rabbit received 20 g. over a period of a month without any sign of ill effects. No external sign of cataract was visible in any of the animals.

### DISCUSSION

Fluorene resembles naphthalene in its metabolism in so far as it is almost completely absorbed in the gut and its metabolites excreted mainly, if not entirely, in the urine. It does not seem to have any marked tendency to produce cataracts although it does appear to form a mercapturic acid derivative (Grantham, Weisburger and Weisburger, 1962). This is not surprising as the connection between mercapturic acid excretion and cataract formation is not very well established (Williams, 1959*a*). The complete absorption of fluorene in the gut is of interest as the tricyclic hydrocarbons in general do not seem to be so well absorbed. In the case of anthracene about 80 per cent and of phenanthrene and acenaphthene about 5 per cent of the hydrocarbon in the diet was recovered unchanged from the faeces (Williams, 1959*b*).

The small amount of phenol found free in neutral urine indicates that the bulk of the phenol excreted is conjugated. The phenol released on acidification is probably present as phosphate or sulphate esters whilst the large amount of phenol released by glucuronidase indicates that the major phenolic metabolite excreted is the glucuronide. The very low levels of free phenol excreted strongly suggest that the material is entirely in the form of glucuronide or other conjugates on leaving the kidneys. Neish (1948) found a much higher level of free phenol in rabbit urine than has been found in the present work but he stored the collected urine with chloroform which has been shown (Sigma, 1962) to be a powerful activator of glucuronidase. It is quite probable that the levels of phenol he found may be due to hydrolysis of the glucuronide during storage. The sex of the animals used by Neish is not recorded but only male animals were used in the present work in view of the remarkably high $\beta$-glucuronidase activity found by Marsh, Levvy and McAllan (1958) in the female rat preputial (clitoral) gland. Harper (1959*a*) showed that the free phenols found in faeces after metabolism of hydrocarbons were formed by hydrolysis of conjugated material in the gut and it seems probable that most if not all of the free phenol found in urine after metabolism of aromatic hydrocarbons is due to enzymic hydrolysis during storage. The practise of adding chloroform to urine to " preserve " it has been extensively adopted in the past.

The species difference in the site of hydroxylation of aromatic hydrocarbons in rats and rabbits has been observed with a variety of hydrocarbons (Harper, 1959*b*, 1959*c*). In general the rat seems to favour hydroxylation on the carbon atom $\alpha$ to the terminal ring junction whilst the rabbit favours hydroxylation at a site two carbon atoms further around the ring. Harper (1959*b*) has shown how this may be interpreted in terms of an ortho form of binding, of the hydrocarbon to cellular protein, being favoured in the rabbit and a para form in the rat.

The microsomal hydroxylation indicated that the species difference in hydroxylation involved the microsomal enzyme system, presumably two different hydroxylating enzymes being present. The lower activity of the rat microsomes compared with rabbit microsomes confirmed the observations of Harper (private communication) on the hydroxylation of pyrene by liver suspensions.

### SUMMARY

1.—It was found that fluorene was apparently harmless to rats and rabbits, completely absorbed in the gut and its phenolic metabolites excreted mainly if not entirely as conjugates.

2.—The major conjugate was the glucuronide but some acid labile conjugates were also excreted.

3. Fluorene was found to be hydroxylated, mainly if not entirely, in the 2 position in the rabbit but in the rat about 70–80 per cent of hydroxylation took place in the 4 position, the rest taking place in the 2 position.

4.—The liver microsomes appear to hydroxylate *in vitro* the same positions as the intact animal hydroxylates *in vivo*, microsomes from the rabbit being much more active than those from the rat.

5.—The difference in the site of hydroxylation is thought to be due to the presence of two different enzymes hydroxylating aromatic hydrocarbons.

The expenses of this work were defrayed from a block grant by the British Empire Cancer Campaign.

### REFERENCES

DEWHURST, F. AND CALCUTT, G.—(1961) *Nature, Lond.*, **191**, 808.

DIELS, O.—(1901) *Ber. dtsch. chem. Ges.*, **34**, 1758.

FRIEDEL, R. A. AND ORCHIN, M.—(1951) 'Ultra Violet Spectra of Aromatic Compounds'. London (Chapman and Hall).

GRANTHAM, P. H., WEISBURGER, E. K. AND WEISBURGER, J. H.—(1961) *J. org. Chem.*, **20**, 1008.—(1962) *Proc. Amer. Ass. Cancer Res.* (Abstracts), **3**, 371.

HARPER, K. H.—(1959*a*) *Brit. J. Cancer*, **13**, 718.—(1959*b*) *Ibid.*, **13**, 732.—(1959*c*) *Ibid.*, **13**, 746.

HEILBRON, I. AND BUNBURY, H. M.—(1953) 'Dictionary of Organic Compounds'. London (Eyre and Spottiswoode).

MARSH, C. A., LEVVY, G. A. AND MCALLAN, A.—(1958) *Biochem. J.*, **69**, 22.

NEISH, W. J. P.—(1948) *Ibid.*, **43**, 533.

SIGMA—(1962) *Sigmachemical Co. Bulletin*, No. 105 (revised edition), St. Louis, Mo., U.S.A.

WEISBURGER, E. K. AND WEISBURGER, J. H.—(1953) *J. org. Chem.*, **18**, 864.—(1958) *Ibid.*, **23**, 1193.

WILLIAMS, R. T.—(1959*a*) ' Detoxication Mechanisms ', 2nd edition. London (Chapman and Hall), p. 208.—(1959*b*) *Ibid.*, p. 206.